# A Prognostic Model Based on RNA Binding Protein Predicts Clinical Outcomes in Hepatocellular Carcinoma Patients

**DOI:** 10.3389/fonc.2020.613102

**Published:** 2021-02-12

**Authors:** Zhongsong Man, Yongqiang Chen, Lu Gao, Guowei Xei, Quanfu Li, Qian Lu, Jun Yan

**Affiliations:** ^1^ Center of Hepatobiliary Pancreatic Disease, XuZhou Central Hospital, Jiangsu, China; ^2^ Department of Clinical Laboratory, XuZhou Central Hospital, Jiangsu, China; ^3^ Center of Hepatobiliary Pancreatic Disease, Beijing Tsinghua Changgung Hospital, Beijing, China; ^4^ Center of Hepatobiliary Pancreatic Disease, The Affiliated Hospital of Qinghai University, Qinghai, China; ^5^ Center of Hepatobiliary Pancreatic Disease, The Second Hospital, Baoding, China

**Keywords:** prognostic prediction model, hepatocellular carcinoma, RNA binding proteins, overall survival, disease-free survival

## Abstract

Dysregulation of RNA binding proteins (RBPs) is closely associated with tumor events. However, the function of RBPs in hepatocellular carcinoma (HCC) has not been fully elucidated. The RNA sequences and relevant clinical data of HCC were retrieved from the The Cancer Genome Atlas (TCGA) database to identify distinct RBPs. Subsequently, univariate and multivariate cox regression analysis was performed to evaluate the overall survival (OS)-associated RBPs. The expression levels of prognostic RBP genes and survival information were analyzed using a series of bioinformatics tool. A total of 365 samples with 1,542 RBPs were included in this study. One hundred and eighty-seven differently RBPs were screened, including 175 up-regulated and 12 down-regulated. The independent OS-associated RBPs of *NHP2, UPF3B*, and *SMG5* were used to develop a prognostic model. Survival analysis showed that low-risk patients had a significantly longer OS and disease-free survival (DFS) when compared to high-risk patients (*HR*: 2.577, *95% CI*: 1.793–3.704, *P* < 0.001 and *HR*: 1.599, *95% CI*: 1.185–2.159, *P* = 0.001, respectively). The International Cancer Genome Consortium (ICGC) database was used to externally validate the model, and the OS of low-risk patients were found to be longer than that of high-risk patients (*P* < 0.001). The Nomograms of OS and DFS were plotted to help in clinical decision making. These results showed that the model was effective and may help in prognostic stratification of HCC patients. The prognostic prediction model based on RBPs provides new insights for HCC diagnosis and personalized treatment.

## Introduction

Hepatocellular carcinoma (HCC) is the fourth leading cause of cancer-associated mortalities, and the sixth leading among cancer incidences globally (1). Despite the significant improvement in diagnostic and treatment approaches, HCC patients have a low survival rate, which is limited to 5 years (2). Surgical resection of HCC tumors is not only a treatment options, but also a source of histopathologic samples that can be investigated to improve the diagnosis and treatment of HCC patients (3–5). The molecular mechanisms underlying the pathogenesis of HCC have not been fully elucidated. As a result, there is an urgent need for sensitive and targeted therapeutic strategies to mitigate HCC.

RNA binding proteins (RBPs) are pleiotropic proteins that regulate gene expression at the post-transcriptional level by interacting with target RNA modules (6, 7). RBPs are generally recognized as proteins that bind to a variety of RNAs, such as ribosomal RNAs (rRNAs), microRNAs (miRNAs), small nuclear RNAs (snRNAs), non-coding RNAs (ncRNAs), messenger RNAs (mRNAs), small nucleolar RNAs (snoRNAs), and transfer RNAs (tRNAs). Research shows that a total of 1,542 RBP genes, accounting for about 7.5% of all protein-coding genes, have been determined in the human genome (6). Previous studies have shown that RBPs are involved in regulating RNA stability, alternative splicing, modification, location, and translation (8). Furthermore, RBPs directly bind to chromatin to regulate gene expression (9). Abnormal, expression of RBP genes adversely affects alternative splicing, polyadenylation apoptosis, among other physiologic processes of the cell (10, 11). Moreover, RBPs have been implicated in processes that promote tumorigenesis and development (10, 12, 13).

This study, aimed at determining the potential functions and molecular mechanisms of differentially expressed RBPs in tumor and normal tissues. Subsequently, OS-associated RBPs were screened using univariate and multivariate cox regression analysis. Finally, independent survival-associated RBPs were used to establish a prognostic prediction model. This study provides potential biomarkers for the diagnosis and treatment of HCC.

## Materials and Methods

### Retrieval of Relevant Molecular Data

Messenger RNA (mRNA) sequence data and clinical information of 50 healthy and 374 HCC tumor tissues were retrieved form The Cancer Genome Atlas database (TCGA, https://portal.gdc.cancer.gov/, updated Oct. 2019). The mRNA data were juxtaposed with relevant clinicopathological data from the TCGA database. Besides, molecular and prognostic data of 260 HCC patients were retrieved form the International Cancer Genome Consortium data set (ICGC-LICH-PIKEN, https://icgc.org/, updated Apr. 2019). First, we selected the 1,542 RBPs according to a previous study (6). The Limma package in R software (3.6.1. https://www.r-project.org/) based on the negative binomial distribution was used to refine the mRNA data and to identify differentially expressed RBPs (14). The sva package in R remove batch effects of TCGA and ICGC database (15). Differentially expressed RBPs with a count value of 0 genes were excluded while those with a |log2 fold change (FC)|>0.5, and false discovery rate (FDR) < 0.05, were considered up-regulated or down-regulated RBPs. The RBPs that were common in both the TCGA and ICGC were selected for this study.

### The GO and KEGG Pathway Enrichment Analyses

Biological functions of differentially expressed RBPs were determined by Gene Ontology (GO) enrichment and Kyoto Encyclopedia of Genes and Genomes (KEGG) pathway analysis based on the clusterProfiler, and org.Hs.eg.db package. The GO analysis terms included a cellular component (CC), molecular function (MF), and biological process (BP).

### Protein-Protein Interaction Network Construction and Module Screening

Protein-protein Interaction (PPI) network for differentially expressed RBPs was predicted using the search tool for the retrieval of interacting genes (STRING; http://string-db.org) from the online database (16). Cytoscape bioinformatics software was used to visualize molecular interaction networks (17). The molecular complex detection (MCODE) method was used to detect molecular complexes in the PPI, and to identify densely connected regions (18). The criteria for selection were as follows: MCODE scores>5, degree cut-off = 2, node score cut-off = 0.2, max depth = 100, and k-score = 2. In this study, RBPs that we found to be disconnected in the PPI network were excluded.

### Identification of Prognosis-Associated RBPs

The overall survival (OS) associated RBPs in the PPI network were examined by univariate cox regression using R statistical software. Furthermore, the multivariate cox proportional hazards regression models were generated based on race, gender, age, body mass index (BMI), grade, residual, T category, N category, M category, and TNM category. Finally, the independent prognosis-associated RBPs were identified by multivariate cox proportional hazards regression analysis.

### Construction of a Prognostic Model

A prognostic prediction model was developed based on the independent prognosis-associated RBPs expression levels and OS data by R software. The formula used for this model was: risk score = β1*Exp1+β2*Exp2+β3*Exp3+…+βi*Expi (where β, coefficient value; Exp, expression level).

The HCC patient prognosis data were divided into low- and high-risk subgroups based on the cutoff value. Afterward, a log-rank test was used to compare the OS and DFS between the low- and high-risk subgroups. The effectiveness of the developed prognostic model was verified using ICGC database. Thereafter, a nomogram for OS and DFS were plotted.

### Verification of Expression Level of HCC-Associated RBPs and Their Prognostic Significance

The RBPs’ expression level of the constructed prognosis model was analyzed through HCC tissues and their paired normal liver tissues. The prognostic value of the RBPs of the developed model in HCC was verified using Kaplan-Meier analysis.

### Statistical Analysis

Kaplan-Meier survival analysis was performed to the survival the OS and DFS for HCC patients. The log-rank test was used to establish the statistical differences between the low- and high-risk patient groups. The correlation between both clinicopathological and risk-score classification was analyzed using the chi-square test. Statistical analyses were performed using R software version 3.6.1. All statistical analyses were performed from at least two independent samples. *P* < 0.05 was considered statistically significant.

## Results

### Identification of Different RBPs in HCC Patients

**Figure 1:**
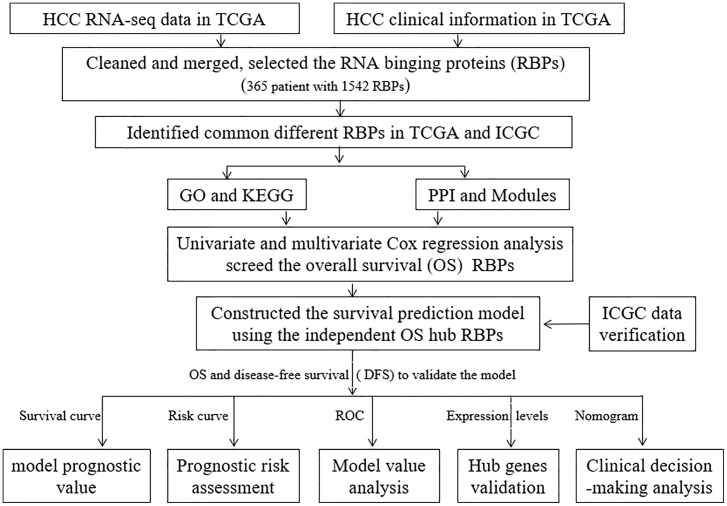
The workflow for this study.

The workflow for this study is shown in **Figure 1**.** **A total of 365 patients (follow-up time (days)>0 (**Supplementary Table 1**) and 1,343 RBPs were included in this study (**Supplementary Table 2**). Out of these, 208 up-regulated and 122 down-regulated differentially expressed RBPs were identified from the TCGA (**Supplementary Table 3**). Eight hundred and 81 up-regulated and 19 down-regulated differentially expressed RBPs were identified from the ICGC data sets (**Supplementary Table 4**). Besides, a total of 187 common differentially expressed (175 up-regulated and 12 down-regulated) RBPs were selected for further analysis (**Supplementary Table 5**).

### The GO and KEGG Enrichment Analysis of the Different RBPs

**Figure 2:**
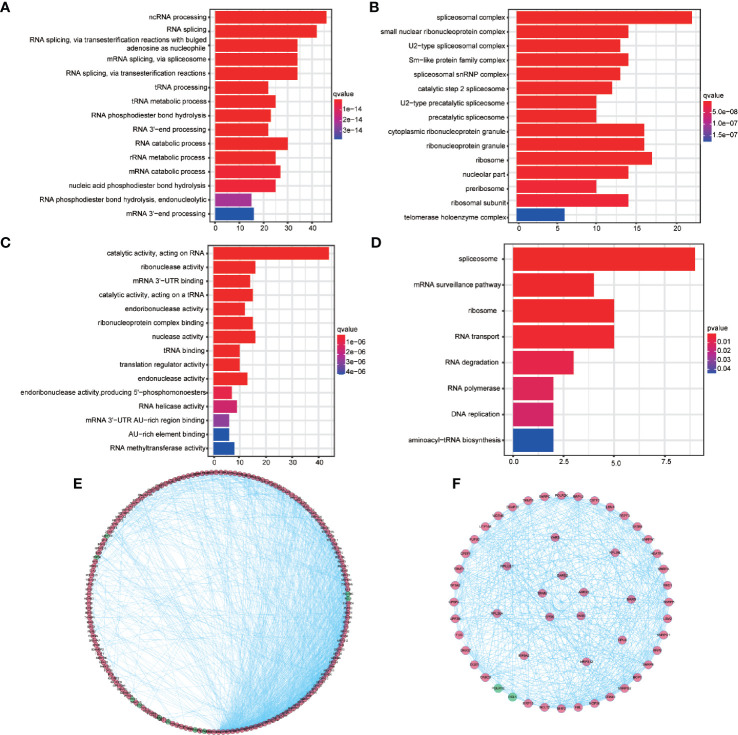
Gene Ontology (GO) and Kyoto Encyclopedia of Genes and Genomes (KEGG) enrichment analysis for 187 differentially expressed RBPs and protein-protein interaction network and modules analysis. **(A)** The top 15 enriched biological process terms for GO. **(B)** The top 15 enriched cellular component terms for GO. **(C)** The top 15 enriched molecular function terms for GO **(D)** The enriched KEGG pathways. **(E)** Protein-protein interaction network of the 187 differentially expressed RBPs. **(F)** Key modules from PPI network. Green circles: down-regulation with a fold change of more than 1.5; red circles: upregulation with fold change of more than 1.5.

A total of 187 common differentially expressed RBPs were performed GO and KEGG enrichment analysis. The 15 highly enriched pathways were showed **Figure 2**. One hundred and eighty-seven RBPs were mainly enriched in the non-coding RNA (ncRNA) processing, RNA splicing, and RNA splicing, through transesterification reactions with bulged adenosine as nucleophile pathways by BP analysis (**Figure 2A**, **Supplementary Table 6**). Furthermore, CC analysis showed that 187 RBPs were enriched in the spliceosomal complex, small nuclear ribonucleoprotein complex and U2-type spliceosomal complex (**Figure 2B**, **Supplementary Table 6**). While for MF, 187 BRPs were enriched in catalytic activity, acting on RNA, ribonuclease activity and mRNA 3′-UTR binding pathways (**Figure 2C**, **Supplementary Table 6**). KEGG enrichment analysis revealed that spliceosome, mRNA surveillance pathway, ribosome, RNA transport, RNA degradation, RNA polymerase, DNA replication, and aminoacyl-transfer RNA (tRNA) biosynthesis were significantly enriched (**Figure 2D**, **Supplementary Table 7**).

### PPI Network Construction and Module Screening

The STRING database network was used to construct the PPI network, which consisted of 187 nodes with an average node degree of 14.3, 283 edges, and a local clustering coefficient of 0.537 (expected number of edges: 357; PPI enrichment *P*-valve: < 1.0e−16). Disconnected nodes were hidden in the network and visualized using the Cytoscape software (**Figure 2E**). Three key modules were obtained from the PPI network using MCODE in Cytoscape (**Figure 2F**, outer, middle, and inner ring, respectively). The conditions for MCODE to identify key modules are as follows: network scoring, degree cutoff: 2 of network scoring; cluster finding, haircut, node density cutoff: 0.1, node score cutoff: 0.2, *k*-core:2, and max. depth: 100. The most significant module genes were found to be enriched in RNA splicing, through transesterification reactions with bulged adenosine as nucleophile, mRNA splicing, through spliceosome pathways and etc. by GO analysis and enriched in spliceosome, mRNA surveillance, ribosome biogenesis in eukaryotes, and RNA polymerase pathways by KEGG analysis (**Supplementary Tables 8** and **9**).

### Identification of Prognosis-Associated RBPs

**Figure 3:**
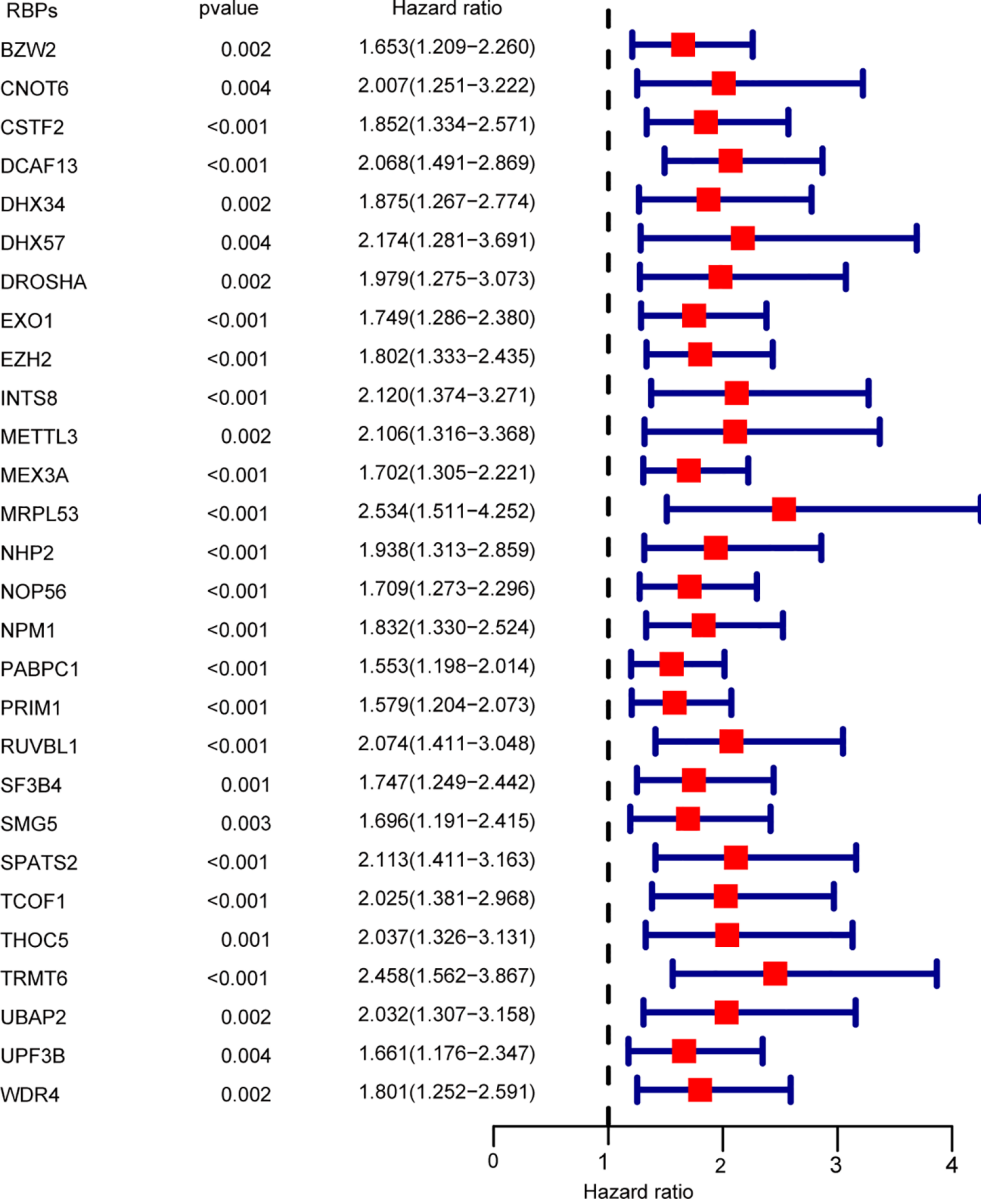
Multivariate cox regression analysis for identification of overall survival (OS)-related RBPs. Multivariate cox regression analysis to identify independent OS-related RBPs.

A total of 93 prognosis-associated RBPs were obtained from univariate cox regression analysis. Subsequently, multivariate cox proportional hazards regression analysis was performed to determine the prognostic value of clinicopathological factors: race, gender, age, body mass index (BMI), tumor residual, tumor grade, T-stage, N-stage, M-stage, and tumor TNM-stage. Twenty-eight independent prognosis-associated RBPs were identified from multivariate cox regression analysis (**Figure 3**).

### Association Between Risk-Score Levels and Clinicopathological Parameters in HCC

Analysis of the relationship between risk-score levels and clinicopathological factors was performed using the chi-square test (**Table 1**). Patients with high risk-scores were positively correlated with tumor grades (*χ2* = 21.844, *P* < 0.001), tumor TNM-stage (*χ2* = 22.603, *P* < 0.001) and T-stage (*χ2* = 22.436, *P* < 0.001), but not with gender (*χ2* = 0.555, *P* = 0.456), age (*χ2* = 0.133, *P* = 0.716), BMI (*χ2* = 2.487, *P* = 0.475), tumor residual (*χ2* = 4.553, *P* = 0.208), N-stage (*χ2* = 2.633, *P* = 0.452), and M-stage (*χ2* = 3.436, *P* = 0.179).

**Table 1:** Relationships between high-, low-risk, and clinicopathological factors.

Factors		Risk-status		*χ ^2^ value*	*P-value*
		Low	High		
Total cases		183	182		
Gender	Female	63	56	0.555	0.456
	Male	120	126		
Age(years)	≤60	85	88	0.133	0.716
	>60	98	94		
BMI	<18.5	8	13	2.487	0.475
	18.5–24.9	78	76		
	≥25	83	74		
Tumor residual	R0	167	153	4.553	0.208
	R1	6	12		
	Rx	7	13		
Tumor grade	G1	38	17	21.844	<0.001
	G2	96	79		
	G3	45	73		
	G4	2	10		
Tumor stage	I	104	66	22.603	<0.001
	II	31	53		
	III	32	51		
	IV	4	0		
T-stage	T1	110	70	22.436	<0.001
	T2	32	59		
	T3	33	45		
	T4	5	8		
N-stage	N0	122	126	2.633	0.452
	N1	1	3		
	Nx	60	52		
M-stage	M0	128	135	3.436	0.179
	M1	3	0		
	Mx	52	47		

### Construction and Validation of a Prognostic Prediction Model

**Figure 4:**
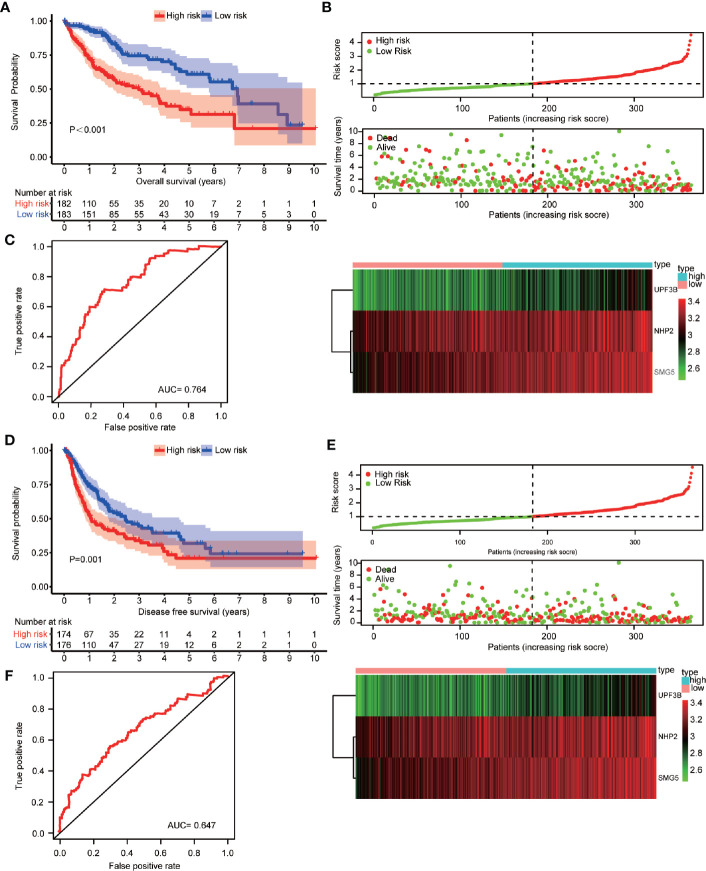
Construction and verification of the prognosis prediction model based on NHP2, UPF3B, and SMG5 and hepatocellular carcinoma (HCC) patients’ overall survival (OS) data. **(A)** Association of risk classification based on NHP2, UPF3B, and SMG5 with OS in HCC patients were showed. Prognosis was depicted with Kaplan-Meier curves: high risk *versus* low risk (low risk: 6.37 *vs*. high risk: 2.981 years; *P* < 0.001). **(B)** Samples were sorted by risk score to investigate the association between the expression levels of NHP2, UPF3B, and SMG5 and risk scores. The risk score for OS of HCC patients positively correlated with the expression of NHP2, UPF3B, and SMG5 (r = 0.556, *P* < 0.001; r = 0.722, *P* < 0.001 and r = 0.828, *P* < 0.001, respectively). The horizontal axis represents the samples and the vertical axis represents risk scores (top cartogram), overall survival (middle cartogram), and expression level (bottom cartogram). **(C)** Receiver operating characteristic (ROC) curves of OS predictors based on NHP2, UPF3B, and SMG5 were showed. **(D)** Association of risk classification based on NHP2, UPF3B, and SMG5 with disease-free survival (DFS) in HCC patients were showed. Prognosis was depicted with Kaplan-Meier curves: high risk *versus* low risk (low risk: 2.447 *vs*. high risk: 1.090 years; *P* = 0.001). **(E)** Samples were sorted by risk score to investigate the association between the expression levels of NHP2, UPF3B, and SMG5 and risk scores. The risk score for DFS of HCC patients positively correlated with the expression of NHP2, UPF3B, and SMG5 (r = 0.556, *P* < 0.001; r = 0.722, *P* < 0.001 and r = 0.828, *P* < 0.001, respectively). The horizontal axis represents the samples and the vertical axis represents risk scores (top cartogram), disease free survival (middle cartogram) and expression level (bottom cartogram). **(F)** ROC curves of DFS predictors based on NHP2, UPF3B, and SMG5 were showed.

A prediction model was constructed based on *NHP2*, *UPF3B*, and *SMG5* expression levels and HCC patients’ OS data. The risk-score of each patient was calculated using the following formula: *Risk score* = 0.399*Exp_NHP2_+0.449*Exp_UPF3B_+0.431*Exp_SMG5_. The cutoff was 1.006528. The patients were distributed into low- or high-risk subgroups by cutoff value (**Supplementary Tables 10** and **11**, respectively). The median OS of HCC patients was 4.641 (*95% CI*: 3.352–5.930) years. Patients in the “low-risk group” (low risk: 6.937 *vs*. high risk: 2.981 years; *P* < 0.001, **Figure 4A**), tumor residual < 1 (residual < 1: 5.074 *vs*. residual≥1: 2.293 years, *P* = 0.007) and TNM-stage < 2 (TNM-stage < 2: 6.937 *vs*. TNM-stage≥2: 3.315 years, *P* < 0.001) were associated with a significant prolonged OS. Patient samples were also sorted based on the risk-score to investigate the association between *NHP2*, *UPF3B*, and *SMG5* expression levels and risk-score (**Figure 4B**). The risk-score was positively correlated with *NHP2*, *UPF3B*, and *SMG5* (r = 0.556, *P* < 0.001; r = 0.722, *P* < 0.001 and r = 0.745, *P* < 0.001, respectively). The AUC of the receiver operating characteristic (ROC) was 0.764 (**Figure 4C**).

Equal results were obtained for patients’ DFS (**Figures 4D–F**). The median DFS of HCC patients was 1.764 (*95% CI*: 1.188–2.341) years. Patients in the “low-risk group” (low risk: 2.447 *vs*. high risk: 1.090 years; *P* = 0.001, **Figure 4D**), tumor residual < 1 (residual < 1: 5.074 *vs*. residual≥1: 2.293 years, *P* = 0.047) and TNM-stage < 2 (TNM-stage < 2: 3.367 *vs*. TNM-stage≥2: 0.953 years, *P* < 0.001) were significantly associated with improved DFS. The risk-score was positively correlated with *NHP2*, *UPF3B*, and *SMG5* expression levels (**Figure 4E**). The AUC was 0.647 (**Figure 4F**).

Multivariate survival analysis, risk score (low-risk and high-risk) (*HR* = 2.110, *95% CI*: 1.359–3.276, *P* = 0.001) and TNM-stage (TNM-stage 1 and TNM-stage 2, 3, 4) (*HR* = 1.773, *95% CI*: 1.168–2.692, *P* = 0.007) were independent predictors of OS in HCC patients (**Table 2**). These results showed that high expression of *NHP2*, *UPF3B*, and *SMG5* is associated with poor prognosis in HCC patients. The high- or low-risk grouping based on the three RBPs may help predict HCC patients’ survival.

**Table 2:** Univariate and multivariate cox regression analysis of clinicopathologic parameters and risk classification for hepatocellular carcinoma (HCC) patients.

		OS				DFS			
		Univariate analysis		Multivariate analysis		Univariate analysis		Multivariate analysis	
Factors		*HR* (*95% CI*)	*P*	*HR* (*95% CI*)	*P*	*HR* (*95% CI*)	*P*	*HR* (*95% CI*)	*P*
Gender	Female	1		1		1		1	
	Male	0.815 (0.572–1.161)	0.257	0.811 (0.533–1.236)	0.330	1.004 (0.731–1.380)	0.970	1.048 (0.729–1.509)	0.799
Age (years)	≤60	1		1		1		1	
	>60	1.249 (0.881–1.771)	0.211	1.010 (0.994–1.027)	0.213	0.985 (0.730–1.329)	0.923	0.925 (0.659–1.300)	0.655
BMI	<18.5	1		1					
	18.5–24.9	1.523 (0.645–3.597)	0.337	1.725 (0.667–4.464)	0.261	1.149 (0.573–2.306)	0.695	1.250 (0.595–2.625)	0.556
	≥25	1.185 (0.503–2.790)	0.698	1.218 (0.467–3.177)	0.686	1.077 (0.539–2.152)	0.833	1.138 (0.536–2.417)	0.736
Tumor residual	R0	1		1		1		1	
	R1 and Rx	2.021 (1.208–3.380)	0.007	1.595 (0.793–3.208)	0.190	1.643 (1.006–2.683)	0.047	2.017 (1.059–3.834)	0.033
Tumor grade	G1–2	1		1		1		1	
	G3–4	1.120 (0.781–1.606)	0.539	1.035 (0.676–1.583)	0.874	1.142 (0.837–1.560)	0.402	1.095 (0.776–1.546)	0.605
Tumor stage	I-II	1		1		1		1	
	III-IV	2.073 (1.418–3.031)	<0.001	1.773 (1.168–2.692)	0.007	2.348 (1.703–3.238)	<0.001	2.225 (1.574–3.146)	<0.001
Risk score	Low	1		1		1		1	
	High	2.577 (1.793–3.704)	<0.001	2.110 (1.359–3.276)	0.001	1.599 (1.185–2.159)	0.001	1.271 (0.901–1.794)	0.172

**Figure 5:**
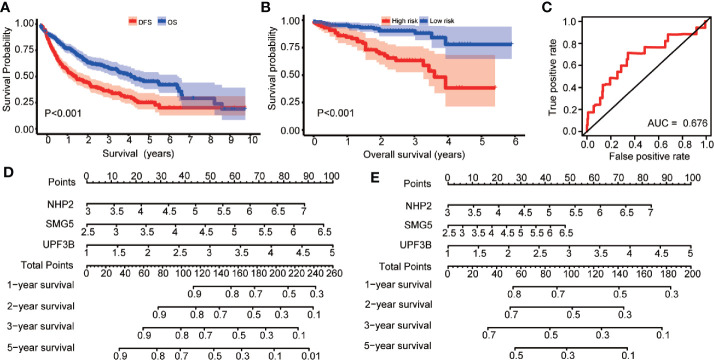
Kaplan-Meier survival analysis are compared the overall survival (OS) and disease-free survival (DFS) curves in hepatocellular carcinoma (HCC) patients; risk score analysis of prognosis prediction model in the International Cancer Genome Consortium (ICGC) cohort; nomogram for predicting 1-, 2-, 3-, and 5-years OS and DFS of HCC patients. **(A)** The Kaplan-Meier survival curve to compare the HCC patients’ survival for OS and DFS in HCC patients. **(B)** Prognosis is depicted with Kaplan-Meier curves for low- and high-risk subgroups in ICGC cohort: high risk *versus* low risk (low risk: >5.221 *vs*. high risk: 3.493 years; *P* < 0.001). **(C)** Receiver operating characteristic (ROC) curves of OS predictors based on NHP2, UPF3B, and SMG5 were showed. **(D)** Nomogram based on the expression of NHP2, UPF3B, and SMG5 for predicting 1-, 2-, 3-, and 5-years OS of HCC patients was showed. **(E)** Nomogram based on the expression of NHP2, UPF3B, and SMG5 for predicting 1-, 2-, 3-, and 5-years DFS of HCC patients was showed.

The 1-, 2-, 3-, and 5-year OS and DFS rates were 83.0, 69.5, 61.5, and 46.9%, and 63.3, 47.7, 38.9, and 24.2%, respectively. Furthermore, OS and DFS curves were compared by Kaplan-Meier survival analysis (**Figure 5A**). The *P*-value was less than 0.001, indicating that it was meaningful to divide OS and DFS for HCC patients. In addition, the external cohort was used to evaluate the survival prediction model. The median OS of HCC patients was 4.651 (*95% CI*: 4.293–5.008) years (**Supplementary Table 12**) and patients in the “high-risk group” were found to have a poor OS than patients in the “low-risk group” in the ICGC database (low risk: >5.221 *vs*. high risk: 3.493 years; *P* < 0.001, **Figure 5B**). Moreover, multivariate survival analysis (adjusted factors: gender, age, and TNM-stage), risk score (low-risk and high-risk) (*HR* = 3.662, *95% CI*: 1.903–7.7047, *P* < 0.001) was independent predictors of OS in HCC patients (**Supplementary Figure S1**). The AUC of the ROC was 0.676 (**Figure 5C**). These results indicated that the model was effective.

### Construction of a Nomogram Based on the Three Hubs RBPs

In this study, a quantitative method for predicting HCC patients’ OS and DFS was developed. This nomogram model for OS and DFS (**Figures 5D, E** **)** was integrated using *NHP2*, *UPF3B*, and *SMG5* and the points were assigned as individual variables. Furthermore, a horizontal line was drawn to determine the point of each RBP variable and the total points for each patient calculated by summing the points of all variables and normalizing it to a distribution from 0 to 100. *NHP2*, *UPF3B*, and *SMG5* were the risk RBPs. By drawing a vertical line between the total point axis and each prognostic axis, each HCC patient’s survival at 1, 2, 3, and 5 years was estimated, which is important in making clinical decisions.

### Validation of Hub RBPs Expression

**Figure 6:**
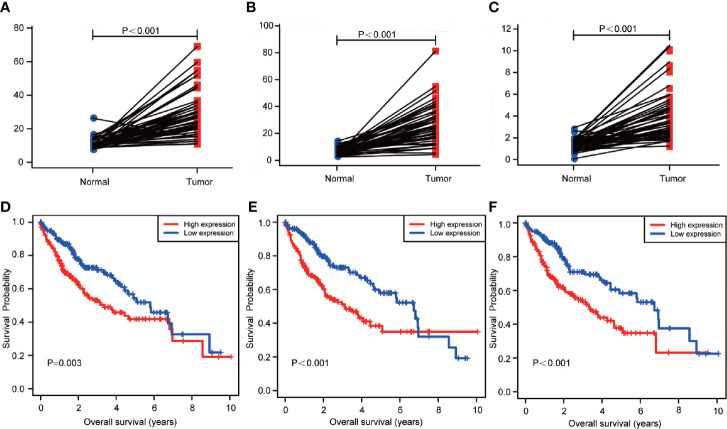
The transcription levels of NHP2, UPF3B, and SMG5 in hepatocellular carcinoma (HCC) and normal liver tissues. And the association with the expression and overall survival (OS) of NHP2, UPF3B, and SMG5 in HCC patients. **(A)** The transcription levels of NHP2 in HCC compared with the paired normal liver tissue was showed (*P* < 0.001). **(B)** The transcription levels of UPF3B in HCC compared with the paired normal liver tissue was showed (*P* < 0.001). **(C)** The transcription levels of SMG5 in HCC compared with the paired normal liver tissue was showed (*P* < 0.001). **(D)** The Kaplan-Meier analysis showed that low expression of NHP2 was associated with improved OS in HCC (P = 0.003). **(E)** The Kaplan-Meier analysis showed that low expression of UPF3B was associated with improved OS in HCC (*P* < 0.001). **(F)** The Kaplan-Meier analysis showed that low expression of SMG5 was associated with improved OS in HCC (*P* < 0.001).

The expression level of *NHP2*, *UPF3B*, and *SMG5* was found to be up-regulated in HCC tissues compared to the paired 50 cases of normal liver tissues in the TCGA database (**Figures 6A–C**). To further explore the prognostic value of the three RBPs in HCC patients, the Kaplan-Meier plotter was used to plot the OS curve for *NHP2*, *UPF3B*, and *SMG5*. The results of the log-rank test showed that the three RBPs were associated with OS in HCC patients (**Figures 6D–F**).

## Discussion

Carcinogenesis is partially mediated by abnormal transcriptional events in the carcinogenic signaling pathways (19). RBPs play a central role in the regulation of gene expression, and their dysregulation has been linked to several human diseases as well as to the occurrence of numerous malignant tumors (20, 21). HCC genome is the imbalance of both coding and non-coding RNA transcriptome (22). In previous study, RBPs are the major mediators of transcriptional changes in HCC carcinogenesis. Gutschner et al. reported RBPs have a broad impact on HCC cell proliferation, survival, and tumor growth (23). A several RBPs have been further studied. eIF3c promoted HCC cell proliferation and tumor growth (24). Gain- and loss-of-function analyses demonstrated that RPS3 promoted HCC tumorigenesis (25).

Previous studies revealed that RBPs are predominantly up-regulated in HCC (26, 27). A total of 1,343 RBPs were included in this study, 784 were highly expressed and while 595 were low-expressed in HCC (**Supplementary Table 2**). Moreover, among the 187 differentially expressed RBPs, 175 were up-regulated while 12 were down-regulated. RBPs are involved in posttranscriptional gene regulation, including cell differentiation, proliferation and cell fate transition. In this study, the different RBPs were significantly enriched in RNA splicing and mRNA splicing, mRNA splicing *via* spliceosome, spliceosome, ribosome as well as translation regulator activity pathways as revealed by GO and KEGG enrichment analysis. Besides, the screened the key module genes were also mainly enriched in RNA splicing and spliceosome. The splicing and spliceosome are post-transcriptional processes in the maturation of mRNAs, which can produce mutations to induce hematological malignancies and solid tumors by alternative splicing (21, 28, 29). In this study, *MBNL2* and *SNRPB* were enriched in RNA splicing and mRNA splicing, and are Fox-dependent elements on alternative splicing of genes involved in tumorigenesis (30). Therefore, we postulated that splicing and spliceosome pathways played a central role in tumorigenesis and the development of RBPs.

In this study, 28 independent prognosis RBPs were screened by multivariate cox proportional hazards regression analysis (**Figure 3** **).** We found that the expression levels of *NHP2*, *UPF3B*, and *SMG5* were up-regulated in HCC compared to normal liver tissues (**Figures 6A–C** **)**. Kaplan-Meier analysis showed that the high expression of *NHP2*, *UPF3B*, and *SMG5* is associated with poor prognosis for HCC patients (**Figures 6D–F**). The correlation between the three RBPs was further investigated. There was a positive correlation between the three genes: *NHP2* and *UPF3B, NHP2*, and *SMG5*, as well as *UPF3B* and *SMG5* (r = 0.117, *P* = 0.026; r = 0.312, *P* = 0.001; r = 0.418, *P* = 0.002, respectively). *NHP2* belongs to the H/ACA small nucleolar ribonucleoproteins (snoRNPs) family and are involved in rRNA processing and modification, and telomerase reverse transcriptase processes (31, 32). *NHP2* mutations can lead to dyskeratosis congenita, a disease that is clinically characterized by pulmonary fibrosis, cirrhosis, and cancer susceptibility (33). Tang *et al*. shown that NHP2 promoted the proliferation of hepatoma cells overexpressing HBx through activating TERT expression (34). UPF3B governs non-sense-mediated RNA decay, and interacts with other non-sense-mediated RNA decay factors to trigger fast RNA decay (35). The non-sense-mediated RNA decay pathway regulates alternative splicing. Besides, Tavan *et al*. reported that UPF3B was up-regulated in serum samples of cholangiocarcinoma patients compared to benign biliary tract diseases, hence can be regarded as a biomarker for differentiating cholangiocarcinoma from benign biliary tract diseases (36). *SMG5* is involved in non-sense-mediated RNA decay. It is highly expressed and is associated with poor prognosis in gastric cancer (37). We selected *NHP2, UPF3B*, and *SMG5* to construct the survival prediction model. Low-risk HCC patients had a better OS compared to the high-risk patients both in univariate (**Figure 4A**) and multivariate (**Table 2**) survival analysis. The ICGC was used as an external cohort to validate the model. And results showed that the low-risk group patients had a better OS than the high-risk group both in univariate (**Figure 5B**) and multivariate (**Supplementary Figure S1**) analysis. Postoperative HCC treatments significantly affect the OS. However, DFS is a relatively unaffected index, therefore, it is more objective to use it in studying. The prediction model was also used to determine the survival risk for HCC patients with DFS. Similar results were obtained for HCC patients with DFS, whereby, patients in the “low-risk group” were associated with a significantly prolonged DFS time compared to patients in the “high-risk group” (low risk: 2.447 *vs*. high risk: 1.090 years; *P* = 0.001, **Figure 4D**, **Table 2**). These results revealed that the survival prediction model was effective and may be used to evaluate prognosis in HCC patients.

Screening for prognostic-associated targets and construction of survival risk model for HCC patients has been reported in recent years. For example, a six-gene signature (*SQSTM1*, *AHSA1*, *VNN2*, *SMG5*, *SRXN1*, and *GLS*) and an eight-gene signature (*DCAF13*, *FAM163A*, *GPR18*, *LRP10*, *PVRIG*, *S100A9*, *SGCB*, and *TNNI3K*) to predict OS for HCC patients have been reported (38, 39). However, the survival prediction model in our study was considered in OS and DFS. Moreover, clinicopathological factors were also included in developing the model. The correlation between the risk-score classification and clinicopathological parameters was also analyzed (**Table 1**). The nomogram for OS and DFS based on *NHP2, UPF3B*, and *SMG5* was found to be effective for use in preliminary clinical decision-making. This study used a series of bioinformatics and statistical methods to integrate selected RBPs to establish prognostic prediction model for HCC patents. This model can help in predicting the survival and management of HCC patients.

This study had various limitations. First, it was based only on the cancer database (TCGA and ICGC) and, therefore, there is a need to validate the finding using large clinical samples. Second, this study was designed based on a retrospective analysis, therefore, a prospective study should be performed to verify the model. Third, the mechanisms of *NHP2*, *UPF3B*, and *SMG5* in HCC need further elucidation.

In conclusion, this study identified differentially expressed and prognosis-associated RBPs, and used them to construct a prognostic prediction model of HCC. This is the first study to report an RBPs-associated prognostic model for HCC patients. The results may help in clinical decision making and guiding individualized treatment for HCC patients.

## Data Availability Statement

Publicly available data sets were analyzed in this study. These data can be found here: The Cancer Genome Atlas (https://portal.gdc.cancer.gov/); the International Cancer Genome Consortium (https://daco.icgc.org/).

## Ethics Statement

Informed consent and ethical recognition were obtained for all cases in the TCGA and ICGC database.

## Author Contributions

ZM, YC, QLu, QLi, and JY supervised the whole project. ZM and YC designed and performed the data analysis. ZM, LG, and GX collected and reprocessed the data. QLu, QLi, and JY designed and performed the experimental validation. ZM, QLu, and JY interpreted the results and wrote the manuscript. All authors contributed to the article and approved the submitted version.

## Funding

The work was supported by the General Program of Natural Science Foundation of China (81372272).

## Conflict of Interest

The authors declare that the research was conducted in the absence of any commercial or financial relationships that could be construed as a potential conflict of interest.

## Supplementary Material

Supplementary Figure 1 Multivariate cox regression analysis of clinicopathologic parameters and risk classification for HCC patients in ICGC database.
