# Supplementary figures and images for: A Prognostic Model Based on RNA Binding Protein Predicts Clinical Outcomes in Hepatocellular Carcinoma Patients
Source: Front Oncol. 2021 Feb 12;10:613102. doi: 10.3389/fonc.2020.613102 (PMC7907500; doi:10.3389/fonc.2020.613102)

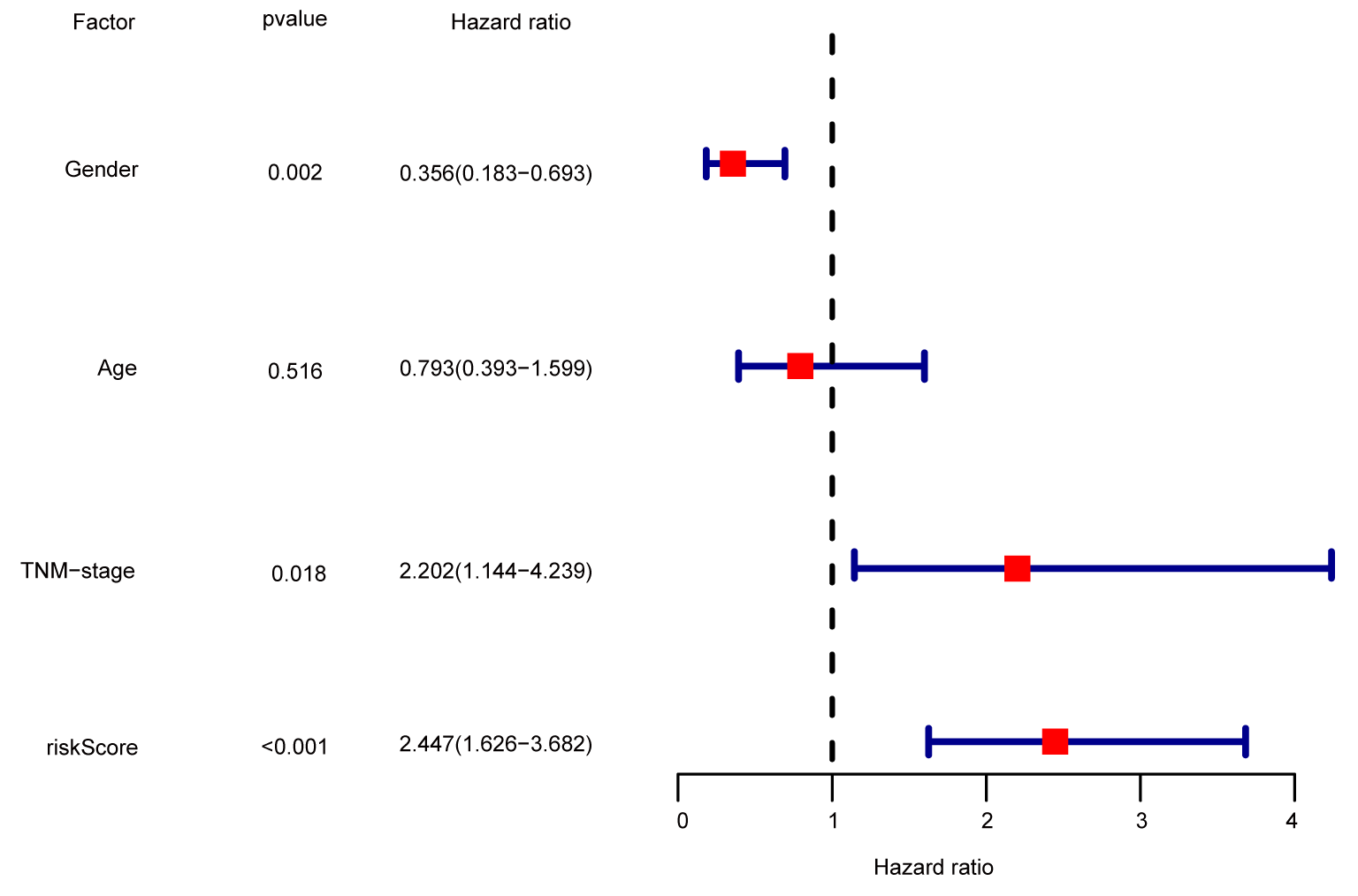

Supplement: Supplementary Figure 1 — Multivariate cox regression analysis of clinicopathologic parameters and risk classification for HCC patients in ICGC database. [file Image_1.tif]
